# Multidimensional Determinants of Obesity: The Role of Life Purpose, Sociodemographics, and Health Habits Across Four Adiposity Scales in a Large Occupational Cohort

**DOI:** 10.3390/medsci13030153

**Published:** 2025-08-25

**Authors:** Pilar García Pertegaz, Pedro Juan Tárraga López, Irene Coll Campayo, Carla Busquets-Cortés, Ángel Arturo López-González, José Ignacio Ramírez-Manent

**Affiliations:** 1 Quirón Salud Palma Planas Hospital, 07010 Palma, Spain; pilarpertegaz@gmail.com; 2 Faculty of Medicine of Castilla la Mancha, 02008 Albacete, Spain; pjtarraga@sescam.jccm.es; 3 ADEMA-Health Group of IUNICS, 07009 Palma, Spain; i.coll@eua.edu.es (I.C.C.); c.busquets@eua.edu.es (C.B.-C.); joseignacio.ramirez@ibsalut.es (J.I.R.-M.); 4 Faculty of Medicine of the Balearic Islands, 07120 Palma, Spain

**Keywords:** obesity, Clínica Universidad de Navarra Body Adiposity Estimator, Metabolic Score for Visceral Fat, life purpose, sociodemographic variables, lifestyle

## Abstract

**Background:** Obesity is multifactorial, shaped by biological, behavioral, and psychosocial factors. Traditional sociodemographic and lifestyle influences are well studied, but psychological well-being, particularly life purpose, remains less explored. This study investigates associations between demographics, health behaviors, and life purpose and obesity prevalence, assessed through four validated adiposity indices in Spanish workers. **Methods:** This cross-sectional study included 93,077 workers (mean age: 43.8 ± 9.6 years; 54.1% men). Obesity was defined according to four measures: body mass index (BMI), waist-to-height ratio (WtHR), Clínica Universidad de Navarra Body Adiposity Estimator (CUN-BAE), and Metabolic Score for Visceral Fat (METS-VF). Sociodemographic, lifestyle (Mediterranean diet, physical activity, smoking), and psychological (Purpose in Life Test, PIL-10) variables were assessed. Logistic regression was used to evaluate associations with obesity risk. **Results:** Low life purpose was significantly associated with increased odds of obesity across all indices, particularly for CUN-BAE (OR = 4.58; 95% CI: 3.99–6.28) and BMI (OR = 5.45; 95% CI: 4.71–6.30). Traditional risk factors such as physical inactivity, poor adherence to the Mediterranean diet, older age, a lower social class, and smoking also demonstrated strong associations with higher adiposity levels. METS-VF showed the greatest sensitivity to male sex and unhealthy behaviors. **Conclusions:** This study identifies life purpose as an independent psychosocial determinant of obesity. Using multiple adiposity measures strengthens the findings, emphasizing psychological well-being in prevention. Longitudinal research is needed to confirm causality and develop interventions enhancing life purpose to improve cardiometabolic health. Given the cross-sectional design, causal inferences cannot be drawn and the directionality of associations remains uncertain.

## 1. Introduction

Obesity is a multifactorial chronic disease characterized by excessive or abnormal fat accumulation, posing a major threat to global public health. The World Health Organization estimates that more than one billion individuals worldwide are currently living with overweight or obesity, conditions that substantially contribute to the global burden of disease [1,2] by increasing the risks of cardiovascular disease (CVD), type 2 diabetes mellitus (T2DM), cancer, musculoskeletal disorders, and premature mortality [3,4,5,6,7,8,9]. The pathophysiology of obesity involves a complex interplay between genetic predisposition, neuroendocrine dysfunction, energy imbalance, and environmental and behavioral factors [10,11,12,13]. Visceral fat functions as an endocrine organ, releasing factors that drive inflammation, insulin resistance, and metabolic dysfunction [14,15].

While body mass index (BMI) remains the most widely used metric to define obesity in both epidemiologic and clinical settings, it has important limitations. BMI does not distinguish between fat and lean mass, nor does it provide information on fat distribution, often leading to misclassification—particularly among individuals with sarcopenic obesity or normal-weight central adiposity [16,17]. As a result, the reliance solely on BMI has been increasingly questioned, and there is growing advocacy for complementary or alternative indicators that better capture cardiometabolic risk.

In recent years, several validated surrogate indices have been proposed to assess both general and visceral adiposity. The waist-to-height ratio (WtHR), a straightforward anthropometric indicator, has demonstrated greater predictive accuracy for cardiometabolic risk than BMI, with a cut-off value of 0.5 widely applied to define central obesity [18,19]. The Clínica Universidad de Navarra Body Adiposity Estimator (CUN-BAE), which incorporates age, sex, and BMI in a nonlinear model, offers an improved estimate of the body fat percentage and has been validated against dual-energy X-ray absorptiometry (DEXA) [20,21]. More recently, the Metabolic Score for Visceral Fat (METS-VF), which integrates age, sex, WtHR, fasting glucose, and triglycerides, has emerged as a robust index for assessing visceral adiposity. The METS-VF index has shown strong predictive abilities for type 2 diabetes mellitus (T2DM), nonalcoholic fatty liver disease (NAFLD), hypertension, stroke, and all-cause mortality, with area under the curve (AUC) values between 0.87 and 0.94 reported in multiple validation studies [22,23,24,25,26].

The clinical relevance of these alternative indices lies in their improved ability to stratify metabolic risk, particularly in occupational or general populations where standard measures may underestimate true adiposity. Studies in Spanish cohorts have reported substantial discrepancies between indices, with the obesity prevalence nearly doubling when defined by CUN-BAE or METS-VF compared to BMI alone [27,28]. Such discrepancies have implications for population-level health surveillance and resource allocation.

Beyond physiological and behavioral influences, psychosocial factors significantly shape obesity risk. One key element is purpose in life, reflecting meaning, direction, and intentionality. Grounded in existential and positive psychology, it has been measured using validated tools, including the Ryff Scales, the Purpose in Life test, and single-item assessments applied in large epidemiologic surveys such as the Health and Retirement Study [29,30].

Higher life purpose correlates with reduced mortality, cardiovascular events, functional decline, and cognitive impairment [31,32,33]. Mechanisms include healthier lifestyles, improved stress regulation, and reduced inflammation and cortisol [34,35]. In contrast, low purpose relates to sedentary behavior, poor diet, smoking, and sleep disturbances, all promoting weight gain and metabolic dysregulation [36,37,38]. Thus, purpose in life emerges as a protective psychosocial factor influencing health outcomes.

Within the framework of obesity research, recent evidence indicates that a low sense of life purpose is independently linked to a higher BMI, greater central adiposity, and an elevated risk of metabolic syndrome, even after controlling for sociodemographic and behavioral confounders [39,40,41]. These observations reinforce the value of incorporating psychological well-being measures into comprehensive models of obesity risk.

Despite this growing interest, large-scale population studies examining the interplay between life purpose, sociodemographic factors, health behaviors, and adiposity indices remain scarce, particularly in occupational settings. Understanding how these variables intersect can inform holistic prevention strategies that transcend conventional lifestyle interventions.

Accordingly, this study aims to examine the relationships between life purpose, sociodemographic factors, and health behaviors (diet, physical activity, and smoking) and obesity prevalence, defined using four validated indices—BMI, WtHR, CUN-BAE, and METS-VF—in a large cohort of Spanish workers. Adopting a multidimensional perspective, it seeks to identify determinants of obesity that extend beyond traditional models, underscoring the potential role of life purpose as a target for public and occupational health interventions.

We hypothesized that higher purpose in life would be inversely associated with obesity prevalence across four adiposity indices, independently of sociodemographic and lifestyle factors.

## 2. Methods

### 2.1. Study Design and Population

This cross-sectional analysis was carried out among Spanish workers participating in a nationwide occupational health surveillance program between January 2019 and December 2023. Participants underwent standardized clinical assessments and completed validated questionnaires in accredited occupational health centers across multiple regions. The study followed the ethical standards of the Declaration of Helsinki and received approval from the corresponding Institutional Ethics Committee. All participants provided written informed consent before inclusion.

### 2.2. Inclusion and Exclusion Criteria

**Figure 1:**
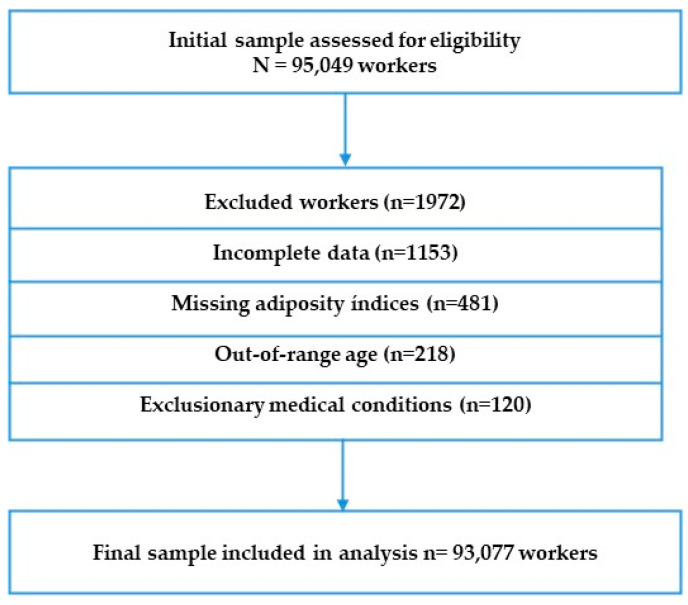
Flow chart of participant selection.

Eligible participants were adults aged 20 to 69 years, employed in any sector and having complete data on anthropometric measurements, biochemical markers, and questionnaire responses. Individuals were excluded if they were pregnant, had known metabolic or endocrine disorders (e.g., thyroid dysfunction, cancer, severe renal impairment), were missing critical data for adiposity indices, or had duplicate or inconsistent records (Figure 1). These criteria align with those previously applied in similar occupational cohort studies in Spain and Europe.

### 2.3. Assessment of Adiposity

Four validated adiposity indices were used to classify obesity:

1. Body Mass Index (BMI)**:** calculated as weight (kg) divided by height squared (m^2^), with obesity defined as BMI ≥ 30 kg/m^2^.
2. Waist-to-Height Ratio (WtHR)**:** waist circumference (cm) divided by height (cm), with a threshold of 0.50 used to define central adiposity [42].
3. Clínica Universidad de Navarra Body Adiposity Estimator (CUN-BAE): a regression formula incorporating age, sex, and BMI to estimate body fat percentage; values ≥ 35% were considered indicative of obesity [43].
4. Metabolic Score for Visceral Fat (METS-VF)**:** calculated using age, sex, WtHR, triglycerides, and fasting glucose; a cut-off of ≥6.3 defined high visceral adiposity. The METS-VF threshold of ≥6.3 has been validated in Spanish and Mediterranean populations [44].

### 2.4. Anthropometric Measuraments

Anthropometric data were obtained by trained personnel using calibrated equipment and standardized protocols. Biochemical parameters, including fasting glucose and triglycerides, were determined through venous blood samples analyzed via enzymatic colorimetric assays in certified laboratories.

Anthropometric variables (height, weight, and waist circumference) were measured according to the standards of the International Society for the Advancement of Kinanthropometry (ISAK) [45]. Participants were assessed in light clothing without shoes. Body weight and height were obtained using a SECA 700 stadiometer and scale (SECA, Chino, CA, USA), and waist circumference was measured at the midpoint between the lower margin of the last palpable rib and the iliac crest with a non-elastic SECA tape (SECA, Chino, CA, USA).

### 2.5. Clincial Measuraments

Blood pressure was determined with an automated oscillometric device (OMROM M3, OMRON, Osaka, Japan) after participants had remained seated at rest for at least 10 min. Three consecutive readings were taken at one-minute intervals, and the mean value was used for analysis. Hypertension was defined as systolic blood pressure ≥ 140 mmHg, diastolic blood pressure ≥ 90 mmHg, or the current use of antihypertensive medication.

### 2.6. Laboratory Analyses

Fasting venous blood samples (12 h) were collected for biochemical analysis. Plasma glucose, triglycerides, and total cholesterol were measured using standardized enzymatic assays. HDL cholesterol was determined by precipitation methods, and LDL cholesterol was calculated using the Friedewald formula, except when triglyceride levels exceeded 400 mg/dL, in which case direct measurement was performed. All values were expressed in mg/dL. Dyslipidemia was defined as lipid concentrations above reference cut-off points or the use of lipid-lowering medication.

### 2.7. Sociodemographic and Lifestyle Variables

Sociodemographic information included age, sex, and occupational social class. Social class was categorized according to the Clasificación Nacional de Actividades Económicas (CNAE-11), applying the criteria of the Spanish Society of Epidemiology (SEE) for classification into social classes I, II, and III [46]:

Class I: Senior executives, directors, and university-educated professionals;

Class II: Intermediate professionals and self-employed individuals;

Class III: Manual laborers.

Lifestyle variables included the following:

- Mediterranean Diet Adherence: Assessed using the 14-item MEDAS-14 questionnaire, which has been validated in Spanish populations [47]. A score ≥9 indicated high adherence.
- Physical Activity: Assessed using the International Physical Activity Questionnaire—Short Form (IPAQ-SF), a tool extensively validated for epidemiological research. Although this questionnaire does not offer the accuracy of direct physical activity measurement, as is the case with pedometers or new technologies, it continues to be recommended for its high reliability and feasibility in epidemiological studies [48], and it has been used in previous studies with good results in the Spanish population [49]. This is a self-reported survey that captures physical activity over the previous seven days. Participants were classified as having low (3.3 METs), moderate (4.0 METs), or high (8.0 METs) physical activity levels. Reliability: MEDAS-14 (α = 0.76–0.82), IPAQ-SF (test–retest reliability ρ = 0.80), PIL-10 (α = 0.85).
- Smoking Status: Self-reported and categorized as current smoker or non-smoker. Individuals who had smoked at least one cigarette per day (or its equivalent) in the past 30 days, or who had quit smoking within the last 12 months, were classified as smokers. Non-smokers included individuals who had abstained from smoking for at least one year or had never smoked.

### 2.8. Purpose in Life Assessment

The purpose in life construct was assessed using the PIL-10, a 10-item version of the Purpose in Life questionnaire adapted for Spanish-speaking adult populations. The PIL-10 evaluates an individual’s sense of direction, existential fulfillment, and life goals on a seven-point Likert scale. Higher scores reflect stronger perceived life purpose. This instrument has demonstrated solid psychometric properties in occupational cohorts [50].

### 2.9. Statistical Analysis

Descriptive statistics were calculated for all variables. Continuous data were expressed as means and standard deviations, and categorical variables as frequencies and percentages. Between-group comparisons were performed using ANOVA for continuous variables and the chi-squared test for categorical variables.

Multivariate logistic regression models were applied to evaluate the associations between obesity, as defined by each index, and the independent variables: age, sex, occupational class, smoking, Mediterranean diet adherence, physical activity, and life purpose. Odds ratios (OR) with 95% confidence intervals (CI) were calculated. All analyses were performed using IBM SPSS Statistics version 29.0 (IBM Corp., Armonk, NY, USA), and statistical significance was defined as *p* < 0.05. No formal correction for multiple comparisons (e.g., Bonferroni or false discovery rate) was applied.

## 3. Results

Table 1 provides a comprehensive overview of the baseline characteristics for 93,077 Spanish workers, stratified by sex. Significant sex-related differences were found across all clinical variables, with men showing higher values in anthropometric and cardiometabolic measures, including BMI, waist circumference, blood pressure, triglycerides, and glucose. Women, conversely, had higher HDL cholesterol levels and a markedly greater proportion reporting adherence to the Mediterranean diet, regular physical activity, and a high sense of life purpose. These differences underscore the importance of sex-stratified analyses when examining lifestyle and psychosocial correlates of obesity.

Table 2 details the mean values (and SDs) for BMI, WtHR, CUN-BAE, and METS-VF across key demographic, behavioral, and psychosocial strata in men and women. Advancing age was consistently linked to increases in all adiposity indices in both sexes. Individuals engaging in unhealthy behaviors—such as physical inactivity or low adherence to the Mediterranean diet—exhibited substantially higher adiposity values, particularly for CUN-BAE and BMI. A pronounced inverse relationship was observed between life purpose and adiposity, suggesting a potential protective psychosocial effect. These findings underscore the multidimensional nature of obesity and the differential sensitivity of adiposity indices to various determinants.

Table 3 shows the prevalence of obesity or elevated adiposity, as determined by four validated indicators, across various subgroups. Among men and women, the obesity prevalence rose with age and was notably higher in lower social classes, smokers, and those lacking physical activity or healthy dietary habits. Particularly striking were the high prevalence rates observed among individuals with low life purpose, especially for the CUN-BAE and METS-VF indices. These findings reinforce the association between psychosocial well-being and obesity risk and demonstrate the utility of combining traditional and novel adiposity markers to better capture population risk profiles.

Table 4 presents the multivariable-adjusted odds ratios and 95% confidence intervals for obesity or elevated adiposity across the four indices. Life purpose emerged as one of the strongest psychosocial predictors, with individuals reporting low life purpose exhibiting markedly higher odds across all indices, particularly for CUN-BAE (OR = 4.58) and BMI (OR = 5.45). Physical inactivity and non-adherence to the Mediterranean diet were also strongly associated with increased adiposity. While the male sex was inversely associated with BMI and CUN-BAE obesity, it strongly predicted high METS-VF (OR = 10.20). The consistency and strength of the associations validate the relevance of psychosocial and lifestyle variables in explaining obesity disparities in working populations.

**Figure 2:**
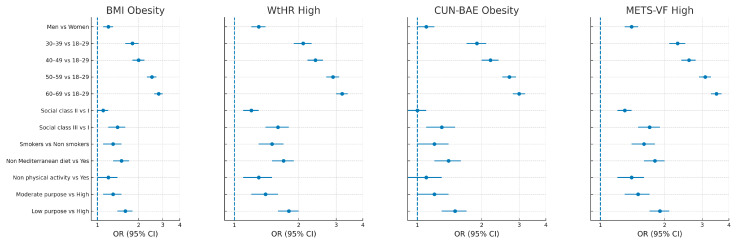
Forest plot.

The forest plot (Figure 2) presented below illustrates the odds ratios (ORs) and 95% confidence intervals (CIs) for various sociodemographic and lifestyle factors associated with four adiposity-related indicators: BMI-defined obesity, elevated waist-to-height ratio (WtHR), CUN-BAE obesity, and high METS-VF. Notably, high METS-VF showed the strongest associations across several subgroups, particularly among physically inactive individuals (OR = 8.96, 95% CI: 7.79–10.14) and those with a low sense of purpose in life (OR = 5.23, 95% CI: 4.38–6.09). While BMI-defined obesity and CUN-BAE presented inverse associations with male sex, WtHR and METS-VF demonstrated markedly higher odds in this group. Consistent age-related trends were observed across all indices, with progressive increases in risk from the youngest to the oldest age groups. These findings underscore the differential sensitivity of adiposity indices to behavioral, psychosocial, and demographic determinants and support the use of multidimensional assessment strategies in occupational and public health settings.

## 4. Discussion

### 4.1. Sociodemographic Determinants

Our study confirmed that sociodemographic variables such as sex, age, education, and occupational class strongly influence obesity risk. Men and older workers exhibited higher obesity prevalences across all adiposity indices, in line with previous Spanish and European occupational studies [51,52]. Lower educational attainment and class III occupations were also linked to greater obesity odds, consistent with evidence that socioeconomic disadvantage promotes obesogenic environments [53,54,55]. These findings underscore the importance of structural and social determinants in shaping obesity risk.

These findings align with national evidence that sociodemographic variables—such as age, education, and income—are significant predictors of BMI in Spain [56,57] and mirror regional disparities by sex and social strata identified at the national level [58].

### 4.2. Behavioral Determinants

Lifestyle behaviors significantly contributed to obesity outcomes. Physical inactivity and smoking were associated with an increased obesity prevalence, supporting the previous literature linking sedentary lifestyles and unhealthy behaviors to metabolic dysregulation [53]. Conversely, adherence to the Mediterranean diet showed a protective role, corroborating prior longitudinal studies demonstrating its beneficial effects on weight control and metabolic health [59]. Moreover, our results concerning the roles of unhealthy dietary patterns and sedentary behaviors are consistent with multivariate analyses showing that poor dietary habits cluster among younger and less educated groups [56,57].

The promotion of the Mediterranean diet not only benefits cardiovascular health but also aligns with climate change mitigation goals, given its lower environmental footprint compared to Western dietary patterns [59,60,61]. These results emphasize that behavioral factors remain modifiable targets for obesity prevention within workplace health programs.

Interestingly, the strongest associations emerged when obesity was defined using METS-VF and CUN-BAE, both of which capture metabolic and visceral fat more accurately than BMI or WtHR alone. This corroborates recent findings from Spanish and Latin American cohorts showing that BMI underestimates true adiposity, particularly in working populations or individuals with metabolically obese–normal weight phenotypes [62,63]. The METS-VF index, incorporating metabolic variables, may be more sensitive in detecting visceral obesity and its related complications, aligning with recent validation studies linking it to diabetes, NAFLD, and cardiovascular events [64,65,66].

### 4.3. Psychological Determinants

A novel contribution of this study is the consistent association between low life purpose and a higher obesity risk across all indices. While life purpose has been previously linked to all-cause mortality, cardiovascular health, and better aging trajectories [67,68,69,70], its relationship with obesity has been underexplored. This extends previous smaller studies linking psychological well-being with healthier body weight trajectories [71]. The PIL-10, as a validated measure of life purpose, captures motivational and existential dimensions that may influence lifestyle choices, stress coping, and long-term health outcomes. Our findings suggest that psychological constructs such as purpose in life should be integrated into obesity research and interventions, particularly in occupational settings, where stress and routine may undermine well-being.

Although literature specifically linking life purpose and obesity remains scarce, evidence suggests that having a strong sense of purpose promotes increased physical activity—as shown by Hooker et al. (2016)—and psychological well-being constructs related to autonomous motivation and health behaviors [72]. Additionally, longitudinal findings on psychosocial stress and weight gain (Fogelman et al., 2022) [73] underscore the relevance of integrating psychological determinants in obesity prevention.

This is consistent with theories suggesting that psychological well-being promotes greater self-regulation, goal-directed behavior, and health responsibility, which protect against obesogenic patterns. Conversely, individuals with low life purpose may experience elevated stress, disinhibition, or neglect of health-related goals, increasing the likelihood of weight gain and visceral fat accumulation. Such mechanisms are supported by evidence linking chronic stress and psychosocial burden with dysregulated cortisol secretion, systemic inflammation, and adiposity [74,75]. A study by Fuentes et al. [76] showed that low psychological well-being is consistently associated with obesity and abdominal fat, suggesting overlapping biological and behavioral pathways.

### 4.4. Interpretation and Implications

From a mechanistic standpoint, life purpose may influence obesity risk through both direct physiological pathways and indirect behavioral mediators. Greater purpose in life has been associated with lower inflammatory markers (e.g., IL-6, CRP), improved glucose metabolism, and favorable neuroendocrine profiles (e.g., lower cortisol) [77,78]. Moreover, individuals with high life purpose are more likely to engage in preventive behaviors, maintain long-term goals, and adhere to healthy routines, which collectively counteract obesity-promoting environments.

In occupational settings, this psychosocial construct may be particularly important, as workers with low engagement, chronic stress, or existential dissatisfaction may be more vulnerable to sedentary lifestyles, emotional eating, or unhealthy coping strategies.

Reverse causation is possible, whereby higher adiposity may reduce psychological well-being. Common method bias from self-reported data could also inflate the observed associations.

Our findings underscore the relevance of integrating mental and existential well-being into workplace health promotion programs.

### 4.5. Strengths and Limitations

This study has several notable strengths. First, it uses a large and diverse occupational cohort with detailed sociodemographic, behavioral, and psychological data, allowing for robust multivariable analyses. Second, it simultaneously applies four validated obesity indices, enabling a nuanced assessment of general and visceral adiposity. Third, the use of the PIL-10 questionnaire provides a validated and culturally adapted measure of life purpose, rarely included in epidemiological studies of obesity.

Nevertheless, certain limitations must be acknowledged. The cross-sectional nature of our study precludes the establishment of temporal or causal relationships between purpose in life and adiposity; associations should be interpreted with caution. Longitudinal studies are required to assess the directionality of associations between life purpose and obesity. Moreover, lifestyle factors and purpose in life were self-reported, introducing potential recall and social desirability biases that could lead to misclassification. Although the PIL-10 is validated, psychological constructs are complex and multifactorial, and residual confounding cannot be excluded. Importantly, there is a scarcity of previous studies directly examining life purpose and obesity risk, particularly using metabolic indices such as the METS-VF or CUN-BAE, which limits direct comparisons.

Another limitation is that our sample consisted exclusively of employed individuals, which may reflect a ‘healthy worker’ effect and limit the generalizability to unemployed, retired, or non-Spanish populations.

Regarding self-reported lifestyle data, although validated tools were employed, responses in working adult populations remain susceptible to recall and social desirability biases.

The large number of statistical tests without type I error adjustment may increase the risk of false-positive findings.

### 4.6. Main Contributions

To our knowledge, this is one of the first studies to systematically evaluate the association between purpose in life and obesity as defined by four different indices in a large working population. It highlights the added value of including psychological well-being in obesity research and prevention strategies, especially in occupational health settings. Our findings suggest that purpose in life is not merely a philosophical or existential concern but a measurable and modifiable determinant of physical health, with significant implications for obesity prevention and chronic disease management.

### 4.7. Future Directions

Future research should explore the longitudinal relationship between life purpose trajectories and changes in body composition, including visceral adiposity. Interventional studies are also needed to determine whether promoting psychological well-being and existential goals can effectively reduce obesity risk or enhance the success of lifestyle interventions. Moreover, integrating purpose-driven components into workplace wellness programs could provide a holistic approach to obesity prevention, addressing not only behavioral risk factors but also meaning and motivation, which may sustain long-term change. Finally, the inclusion of objective biomarkers of stress and inflammation, along with neuroimaging and behavioral data, could further elucidate the biological pathways linking life purpose to adiposity and metabolic health.

## 5. Conclusions

Our findings highlight that life purpose is a novel and independent determinant of obesity among Spanish workers. Alongside established sociodemographic and lifestyle predictors, a low sense of purpose was consistently associated with a higher obesity prevalence across multiple adiposity indices. These results suggest that occupational health interventions should not only target lifestyle modification but also incorporate psychological well-being strategies. Strengthening life purpose in the working population may represent an innovative approach to obesity prevention and management.

Given the paucity of previous studies examining the relationship between life purpose and obesity, this research fills an important gap and opens new avenues for interdisciplinary exploration. Future longitudinal and intervention studies are needed to elucidate causal pathways and to assess whether fostering life purpose could serve as an effective strategy for obesity prevention and the promotion of metabolic health.

## Figures and Tables

**Table 1:** Sociodemographic, clinical, and lifestyle characteristics of the study population by sex.

	Men *n* = 55,900	Women *n* = 37,177	
Variable	Mean (SD)	Mean (SD)	*p*-value
Age (years)	39.8 (10.3)	39.3 (10.2)	<0.001
Height (cm)	174.0 (7.0)	161.2 (6.6)	<0.001
Weight (kg)	81.2 (13.9)	65.4 (13.2)	<0.001
Waist (cm)	87.7 (9.1)	73.9 (7.9)	<0.001
Hip (cm)	100.1 (8.4)	97.3 (8.9)	<0.001
Systolic BP (mm Hg)	124.3 (14.9)	114.5 (15.0)	<0.001
Diastolic BP (mm Hg)	75.4 (10.6)	69.7 (10.4)	<0.001
Cholesterol (mg/dL)	195.9 (38.8)	193.5 (36.4)	<0.001
HDL-c (mg/dL)	51.0 (7.1)	53.8 (7.7)	<0.001
LDL-c (mg/dL)	120.5 (37.7)	122.1 (37.0)	<0.001
Triglycerides (mg/dL)	123.7 (87.7)	88.5 (47.2)	<0.001
Glucose (mg/dL)	88.1 (13.0)	84.1 (11.5)	<0.001
Variable	*n* (%)	*n* (%)	*p*-value
18–29 years	9956 (17.8)	7193 (19.3)	<0.001
30–39 years	18,525 (33.1)	12319 (33.1)	
40–49 years	16,632 (29.8)	11,035 (29.7)	
50–59 years	9062 (16.2)	5669 (15.2)	
60–69 years	1725 (3.1)	961 (2.6)	
Social class I	2964 (5.3)	2587 (7.0)	<0.001
Social class II	9702 (17.4)	12,197 (32.8)	
Social class III	43,234 (77.3)	22,393 (60.2)	
Smokers	20,659 (37.0)	12,262 (33.0)	<0.001
Yes, Mediterranean diet	22,838 (40.9)	19,096 (51.4)	<0.001
Yes, physical activity	25,285 (45.2)	19,337 (52.0)	<0.001
Purpose in life, low	19,071 (34.1)	4432 (11.9)	<0.001
Purpose in life, moderate	27,707 (49.6)	13,774 (37.0)	
Purpose in life, high	9122 (16.3)	18,971 (51.0)	

BP, blood pressure. HDL, high-density lipoprotein. LDL, low-density lipoprotein. SD, standard deviation.

**Table 2:** Mean values of four adiposity indices according to sociodemographic, behavioral, and psychosocial factors, stratified by sex.

		BMI	WtHR	CUN-BAE	METS-VF
Men	*n*	Mean (SD)	Mean (SD)	Mean (SD)	Mean (SD)
18–29 years	9956	25.0 (4.1)	0.49 (0.05)	21.1 (6.3)	5.9 (0.5)
30–39 years	18,525	26.5 (4.1)	0.50 (0.05)	24.6 (5.8)	6.3 (0.5)
40–49 years	16,632	27.4 (4.1)	0.52 (0.05)	27.2 (5.4)	6.6 (0.5)
50–59 years	9062	28.0 (4.1)	0.53 (0.05)	29.0 (5.1)	6.8 (0.5)
60–69 years	1725	28.4 (3.8)	0.54 (0.05)	30.4 (4.5)	6.9 (0.4)
Social class I	2964	26.6 (3.8)	0,50 (0.05)	25.5 (5.7)	6.4 (0.5)
Social class II	9702	26.7 (4.0)	0.51 (0.05)	25.5 (6.0)	6.4 (0.6)
Social class III	43,234	26.9 (4.3)	0.51 (0.05)	25.7 (6.3)	6.5 (0.6)
Smokers	20,659	27.2 (4.1)	0.51 (0.05)	26.2 (6.0)	6.5 (0.6)
Non-smokers	35,241	26.2 (4.3)	0.50 (0.05)	24.6 (6.5)	6.4 (0.6)
Yes, Mediterranean diet	22,838	24.0 (2.2)	0.48 (0.03)	21.2 (4.1)	6.1 (0.5)
Non-Mediterranean diet	33,062	28.7 (4.2)	0.53 (0.05)	28.7 (5.7)	6.7 (0.5)
Yes, physical activity	25,285	24.0 (2.2)	0.48 (0.03)	21.3 (4.1)	6.1 (0.5)
Non-physical activity	30,615	29.1 (4.1)	0.53 (0.05)	29.2 (5.4)	6.7 (0.5)
Purpose in life, low	19,071	29.2 (4.7)	0.54 (0.05)	29.9 (5.7)	6.9 (0.4)
Purpose in life, moderate	27,707	26.1 (3.3)	0.50 (0.04)	24.6 (5.0)	6.3 (0.5)
Purpose in life, high	9122	24.0 (2.9)	0.47 (0.03)	20.0 (4.9)	5.8 (0.4)
Women	*n*	Mean (SD)	Mean (SD)	Mean (SD)	Mean (SD)
18–29 years	7193	23.8 (4.8)	0.44 (0.05)	31.2 (6.9)	5.0 (0.7)
30–39 years	12,319	24.6 (4.9)	0.45 (0.05)	33.8 (6.7)	5.3 (0.7)
40–49 years	11,035	25.7 (4.8)	0.46 (0.05)	36.7 (6.1)	5.6 (0.7)
50–59 years	5669	26.7 (4.7)	0.47 (0.05)	39.3 (5.4)	5.9 (0.6)
60–69 years	961	27.5 (4.4)	0.48 (0.05)	41.2 (4.7)	6.1 (0.6)
Social class I	2587	24.0 (4.4)	0.45 (0.05)	33.3 (6.3)	5.2 (0.7)
Social class II	12,197	24.1 (4.5)	0.45 (0.05)	33.6 (6.4)	5.3 (0.7)
Social class III	22,393	25.9 (5.1)	0.47 (0.05)	36.3 (7.0)	5.6 (0.7)
Smokers	12,262	25.5 (5.0)	0.46 (0.05)	35.7 (6.9)	5.5 (0.7)
Non-smokers	24,915	24.5 (4.8)	0.45 (0.05)	34.1 (6.8)	5.4 (0.8)
Yes, Mediterranean diet	19,096	22.4 (2.4)	0.44 (0.04)	31.2 (4.3)	5.1 (0.6)
Non-Mediterranean diet	18,081	28.1 (5.3)	0.48 (0.05)	39.4 (6.6)	5.8 (0.7)
Yes, physical activity	19,337	22.3 (2.3)	0.44 (0.04)	30.9 (4.2)	5.1 (0.6)
Non-physical activity	17,840	28.3 (5.1)	0.48 (0.05)	39.8 (6.2)	5.9 (0.6)
Purpose in life, low	4432	32.1 (6.5)	0.52 (0.06)	44.4 (6.4)	6.4 (0.5)
Purpose in life, moderate	13,774	24.9 (4.2)	0.46 (0.05)	35.2 (6.2)	5.5 (0.7)
Purpose in life, high	18,971	23.8 (3.5)	0.44 (0.04)	33.1 (5.6)	5.2 (0.7)

BMI, body mass index. WtHR, waist-to-height ratio. CUN-BAE, Clinica Universitaria de Navarra Body Adiposity Estimator. METS-VF, Metabolic Score for Visceral Fat.

**Table 3:** Prevalence of obesity and high adiposity according to four indicators and sociodemographic, lifestyle, and psychosocial variables, stratified by sex.

		BMI Obesity	WtHR High	CUN-BAE Obesity	METS-VF High
Men	*n*	%	%	%	%
18–29 years	9956	10.9	31.8	22.9	0.6
30–39 years	18,525	16.5	43.3	43.4	3.5
40–49 years	16,632	23.0	53.6	64.0	11.4
50–59 years	9062	27.7	61.2	79.1	20.3
60–69 years	1725	30.5	68.2	89.9	30.8
Social class I	2964	17.4	44.6	51.3	7.9
Social class II	9702	20.4	45.6	52.3	8.0
Social class III	43,234	26.5	48.9	53.5	9.2
Smokers	20,659	21.3	50.4	57.0	9.0
Non-smokers	35,241	16.9	44.0	46.5	8.9
Yes, Mediterranean diet	22,838	6.5	24.8	19.3	4.8
Non-Mediterranean diet	33,062	23.2	64.0	76.5	10.3
Yes, physical activity	25,285	6.0	24.1	19.7	3.1
Non-physical activity	30,615	27.5	67.1	80.7	14.3
Purpose in life, low	19,071	37.2	75.9	81.5	16.2
Purpose in life, moderate	27,707	13.1	40.3	46.3	5.9
Purpose in life, high	9122	3.1	13.3	14.4	1.2
Women	*n*	%	%	%	%
18–29 years	7193	10.5	11.1	24.3	0.1
30–39 years	12,319	13.2	13.7	36.2	0.3
40–49 years	11,035	16.9	17.8	55.3	0.5
50–59 years	5669	21.6	22.7	77.8	1.3
60–69 years	961	25.9	26.7	91.5	1.7
Social class I	2587	9.7	10.4	35.1	0.3
Social class II	12,197	10.0	11.1	35.6	0.7
Social class III	22,393	19.1	19.5	55.1	0.9
Smokers	12,262	17.0	16.8	50.5	0.5
Non-smokers	24,915	12.2	14.6	40.9	0.4
Yes, Mediterranean diet	19,096	6.2	7.5	21.4	0.3
Non-Mediterranean diet	18,081	21.1	30.3	74.7	0.9
Yes, physical activity	19,337	5.5	6.8	18.1	0.2
Non-physical activity	17,840	26.3	30.5	79.1	1.1
Purpose in life, low	4432	60.3	66.5	91.6	3.0
Purpose in life, moderate	13,774	13.9	18.2	49.7	1.3
Purpose in life, high	18,971	6.0	6.9	35.3	0.6

BMI, body mass index. WtHR, waist-to-height ratio. CUN-BAE, Clinica Universitaria de Navarra Body Adiposity Estimator. METS-VF, Metabolic Score for Visceral Fat.

**Table 4:** Multivariate odds ratios for obesity or high adiposity by four indices according to sociodemographic, behavioral, and psychosocial predictors.

	BMI Obesity	WtHR High	CUN-BAE Obesity	METS-VF High
	OR (95% CI)	OR (95% CI)	OR (95% CI)	OR (95% CI)
Women	1	1	1	1
Men	0.90 (0.86–0.94)	2.41 (2.31–2.51)	0.87 (0.83–0.91)	10.20 (8.76–11.65)
18–29 years	1	1	1	1
30–39 years	1.41 (1.34–1.48)	1.36 (1.30–1.43)	1.53 (1.39–1.67)	1.29 (1.20–1.39)
40–49 years	1.89 (1.72–2.07)	1.72 (1.63–1.82)	2.03 (1.75–2.31)	1.52 (1.40–1.65)
50–59 years	2.29 (1.99–2.59)	2.39 (2.28–2.50)	3.00 (2.58–3.43)	1.93 (1.78–2.09)
60–69 years	3.85 (3.36–4.35)	3.69 (3.40–4.00)	4.46 (3.81–5.12)	3.28 (2.78–3.79)
Social class I	1	1	1	1
Social class II	1.39 (1.30–1.49)	1.31 (1.22–1.40)	1.34 (1.25–1.44)	1.18 (1.14–1.23)
Social class III	1.72 (1.57–1.88)	1.66 (1.50–1.83)	1.54 (1.42–1.66)	1.36 (1.29–1.44)
Non-smokers	1	1	1	1
Smokers	1.12 (1.09–1.15)	1.21 (1.14–1.29)	1.26 (1.20–1.33)	1.21 (1.17–1.26)
Yes, Mediterranean diet	1	1	1	1
Non-Mediterranean diet	2.57 (2.25–2.89)	1.41 (1.31–1.51)	1.51 (1.42–1.61)	4.51 (3.72–5.32)
Yes, physical activity	1	1	1	1
Non-physical activity	6.16 (5.37–6.96)	3.16 (2.94–3.39)	8.48 (7.19–9.78)	8.96 (7.79–10.14)
Purpose in life, high	1	1	1	1
Purpose in life, moderate	2.37 (2.04–2.70)	1.88 (1.72–2.05)	3.12 (2.70–3.54)	2.69 (2.30–3.09)
Purpose in life, low	5.45 (4.71–6.30)	2.89 (2.59–3.20)	4.58 (3.99–6.28)	5.23 (4.38–6.09)

BMI, body mass index. WtHR, waist-to-height ratio. CUN-BAE, Clinica Universitaria de Navarra Body Adiposity Estimator. METS-VF, Metabolic Score for Visceral Fat. OR, odds ratio. CI, confidence interval.

## Data Availability

The original contributions presented in this study are included in the article material. Further inquiries can be directed to the corresponding author(s).
